# A Dual-Type L2 11-88 Peptide from HPV Types 16/18 Formulated in Montanide ISA 720 Induced Strong and Balanced Th1/Th2 Immune Responses, Associated with High Titers of Broad Spectrum Cross-Reactive Antibodies in Vaccinated Mice

**DOI:** 10.1155/2018/9464186

**Published:** 2018-05-03

**Authors:** Farhad Motavalli Khiavi, Arash Arashkia, Majid Golkar, Maryam Nasimi, Farzin Roohvand, Kayhan Azadmanesh

**Affiliations:** ^1^Department of Virology, Pasteur Institute of Iran, Tehran, Iran; ^2^Department of Parasitology, Pasteur Institute of Iran, Tehran, Iran; ^3^Department of Dermatology, Tehran University of Medical Sciences, Tehran, Iran

## Abstract

*E. coli*-derived concatenated, multitype L2-conserved epitopes of human papillomavirus (HPV) L2 protein might represent a less expensive and pan-type vaccine alternative (compared to type-specific HPV L1 virus-like particles), if stable protein expression and strong immunogenicity features could be met. Herein, three dual-type- (DT-) HPV L2 fusion peptides comprising the three head-to-tail tandem repeats (multimers) of either HPV 16 epitope “17-36” or “69-81” or one copy (monomer) of 11-88 fused to the same residues of HPV 18 were constructed and expressed in *E. coli*. SDS-PAGE and Western blot analyses indicated the proper expression and stability of the *E. coli*-derived DT peptides. Mice immunized by formulation of the purified DT peptides and Freund's adjuvant (CFA/IFA) raised neutralizing antibodies (NAbs; the highest for DT: 11-88 peptide) which showed proper cross-reactivity to HPV types: 18, 16, 31, and 45 and efficiently neutralized HPV 18/16 pseudoviruses *in vitro*. Immunization studies in mice by formulation of the DT: 11-88 × 1 peptide with various adjuvants (alum, MF59, and Montanides ISA 720 and 50) indicated that Montanide adjuvants elicited the highest cross-reactive titers of NAbs and similar levels of IgG1 and IgG2a (switching towards balanced Th1/Th2 responses). The results implied development of low-cost *E. coli*-derived DT: 11-88 peptide formulated in human compatible ISA 720 adjuvant as a HPV vaccine.

## 1. Introduction

Human papillomavirus (HPV), a heterogeneous group of nonenveloped double-stranded DNA tumor viruses with around 8 kb genome encoding for early and late (L1 and L2 capsids) proteins, infects the epithelia of the skin and mucosa in humans. HPV-induced diseases range from subclinical and self-limiting benign skin warts and mucosal papillomas (condylomas) to life-threatening dysplasias and carcinomas of the anogenital tract and oropharynx [1, 2]. To date, more than 200 genotypes of HPV have been identified, among which at least 15 types (16, 18, 31, 33, 35, 39, 45, 51, 52, 56, 58, 59, 68, 73, and 82) are responsible for 5% of global cancer cases (especially cervical cancer) [3–5]. Of note, persistent infection with four HPV types—16, 18, 31, and 45—is suggested to be the cause of more than 90% of global cervical carcinomas [6, 7]. Indeed, worldwide meta-analyses have indicated that two-thirds of women with cervical cancer were infected by either HPV 16 (51%) or HPV 18 (16.2%) genotypes [8]. More recently, a study on HPV prevalence and genotypes in various histological subtypes of cervical adenocarcinoma indicated that HPV 16 was the most common of HPV-positive cases (50.9%), followed by HPV 18 (31.6%) and HPV 45 (11.6%) [9].

Prior studies indicated that neutralizing antibodies (NAbs) to capsid protein L1 confer sterilizing immunity against HPV infection. Although, NAbs are raised against both L1 and L2 (which encode for major and minor capsid proteins, resp.), high titers of NAbs are raised against L1 since B cell epitopes of L1 are highly dominant compared to those of L2 [10]. Accordingly, the capability of heterologously produced recombinant L1 protein to self-assemble into virus-like-particles (L1-VLPs) and induce strong and stable immune response was reported [11]. These findings finally resulted in the development of efficient prophylactic HPV L1-VLPs-based vaccines which were approved for human applications including (i) the quadrivalent Gardasil (containing L1 of types 6/11/16/18 produced in yeast and formulated with aluminum hydroxyphosphate sulfate adjuvant), (ii) bivalent Cervarix (containing L1 of types 16 and 18 produced in insect cells formulated with ASO4 adjuvant) [12], and more recently (iii) a 9-valent Gardasil (containing L1 of types 6/11/16/18/31/33/45/52/58) [13]. Although the 9-valent vaccine has the potential to prevent 85–95% of HPV-related cancers [14], but protection is restricted to the types used in the vaccine preparation and it does not protect against cutaneous HPV types causing benign skin warts or types implicated in the development of nonmelanoma skin cancer (NMSC) in immunosuppressed patients [15]. Another important limitation of L1-based VLP vaccines is the high cost due to the technical complexity of the VLP production process. This limits the access of many developing countries to the vaccine, considering higher incidence rate of infection compared to developed countries (42.4% versus 22.6%) [16]. Therefore, development of a prophylactic HPV vaccine with much lower price and less manufacturing complexity is an urgent need for low-income developing countries [17].

Contrary to immune responses against strong conformational L1-VLP epitopes that are type-specific, the N-terminal of minor capsid protein L2 and amino acids 11-200 (from the total of 473 residues) contain conserved, type-common (pan-type) but subdominant linear B cell epitopes. Thus, these epitopes are capable of inducing broadly cross-NAbs to highly divergent and heterogeneous HPV types, even in the isolated forms, but in much lower titers and potency [18]. Therefore, development of a vaccine formulation based on HPV L2 linear epitopes with increased immunogenicity to elicit strong antibody responses might be an alternative to a complex and highly multivalent HPV L1-VLP vaccine. To this end, several protective type-common HPV L2 epitopic peptides within 11-200 were identified and used in animal immunization studies, among which residues 11-88, 69-81, and 17-36 (RG-1) attracted the highest attentions [19]. Results of these studies indicated that the HPV L2 peptide 11-88 was the most efficient immunogen in induction of cross-clade NAbs, comparable (or even better) to that of 11-200 [20], while reports on epitope 69-81, another pan-type immunodeterminant exposed on the surface of HPV virions, were controversial [21]. In fact, results of earlier studies indicated that VLPs displaying this epitope could induce NAbs to even the distantly related HPV 11 [22]. However, later studies demonstrated that immunization with HPV 16 L2 peptide 69-81 induced strong homologous protection, but little cross-protection against heterologous HPV types. Subsequently, this shortcoming was overcome by immunizing with VLPs displaying HPV L2 65-85 consensus sequence of other high-risk HPV types [23]. Accordingly, the single epitope comprising amino acids 17-36 (initially identified by the mouse neutralizing monoclonal antibody “RG1”) was also shown to be capable of providing broad cross-neutralizing activity against heterologous papillomavirus types, but similar to results of immunization by other HPV L2 peptides, titers of cross-protection were very low [24, 25]. As such, several approaches were subsequently employed to widen the spectrum of protection and enhance the antibody titers for these HPV L2 epitopes. These approaches generally included (i) the use of HPV L1 capsid as a scaffold to make L2-presenting VLPs [26], (ii) displaying on various bacteriophages [23] and viruses [27], and (iii) there are several reports on exploiting different adjuvant formulations to enhance neutralizing antibody responses against L2-based subunit vaccines. In this respect, aluminum (alum) compounds are widely used to boost antibody response induced by L2 peptides in mice [19]. Despite eliciting neutralizing antibodies against L2 peptides such as 11-88 and 17-36, these responses were weak and mainly Th2-biased [28]. Hence, in some studies, a combination of alum and MPL (TLR-4 agonist) or CpG oligo (TLR-9 agonist) was used and showed to be able to induce both antibody and Th1 responses [20, 29]. Furthermore, bacterial flagellin, a TLR-5 agonist, fused to either L2 11-200 or 11-88 × 8 stimulated strong neutralizing antibody response in mice [30]. Montanides are water in oil adjuvants that have been studied in several therapeutic and prophylactic vaccine studies. There are little reports on using Montanides to formulate HPV L2-based vaccines in which appropriate neutralizing antibody responses have been elicited [27].

In still another approach to develop low-cost HPV L2-based vaccines with improved immunogenicity by production in bacterial expression systems and multimeric, concatenated, and epitope-encoding peptides formulated with strong adjuvants was developed [19]. It should be noted that, although, NAbs against L2 cross-neutralize diverse papillomavirus types, Ab titers against the endogenous vaccine-derived antigen are generally higher compared with those of heterologous types, which might limit their application. Accordingly, construction of an antigen composed of concatenated multitype HPV L2 epitopes might broaden the spectrum of the protection towards several clinically relevant HPV genotypes. In this context, immunization studies in mice and rabbit indicated that concatenated RG1-encoding peptides of 22 clinically relevant HPV genotypes (i.e., 17-36 × 22) and fusion peptides of divergent HPV L2 11-88 peptides (11-88 × 8 and 11-88 × 5) or that of 13-47 × 15 peptides (HPV 6, HPV 11, HPV 16, HPV 18, HPV 31, HPV 33, HPV 35, HPV 39, HPV 45, HPV 51, HPV 52, HPV 56, HPV 58, HPV 59, and HPV 73) resulted in induction of pan-HPV NAbs [20, 29]. However, the stability and proper expression of such complex antigen formulations in bacterial systems was a major drawback and limiting factor, as the increased copy number resulted in the protein degradation that critically declined the induction of immune responses [20, 29, 31, 32].

Prior studies indicated that application of three or more tandem repeats of the same epitope (amino acids 20-38) of L2 peptide in the context of linear protein induced more potent immune response in comparison with the same residues in monopeptide form [33]. Since, HPV 16/HPV 31 and HPV 18/HPV 45 pairs are evolutionary closely related to each other [20, 34], it might be expected that NAbs raised against HPV 16/18 L2-peptides should be at least effective against HPV 31/45 too. If so, such an immunogen might have the potential to be used for vaccination against more than 90% of cervical carcinomas as mentioned above [7–9, 35]. Considering these points, we hypothesized that it might be possible to develop a simple and low-cost pan-HPV vaccine (at least effective against types 16, 18, 31, and 45) by designing and stably expressing in bacterial system of dual-type L2 fusion peptides from HPV types 16/18 formulated with a proper adjuvant. To this end, we recently evaluated the immunogenicity of a “dual-type HPV 16/18 L2 fusion (DT) peptide” comprising the three head-to-tail tandem repeats (multimers) of HPV L2 RG-1 epitope “17-36 residues (17-36 × 3)” in murine model with promising results [36]. In the present study, the immunogenicity of three “DT peptides” comprising the three head-to-tail tandem repeats (multimers) of either HPV L2 RG-1 epitope “17-36 residues (17-36 × 3)” or “69-81 residues (69-81 × 3)” or one copy (monomer) of 11-88 amino acids (11-88 × 1) from HPV 16 fused to the same regions, with the same patterns (i.e., 17-36 × 3 or 69-81 × 3 or 11-88 × 1, resp.) of HPV 18, was evaluated in mice and the best immunogen out of the three was further evaluated for best adjuvant formulation.

## 2. Materials and Methods

### 2.1. Generation of the HPV L2-Encoding Constructs

**Figure 1:**
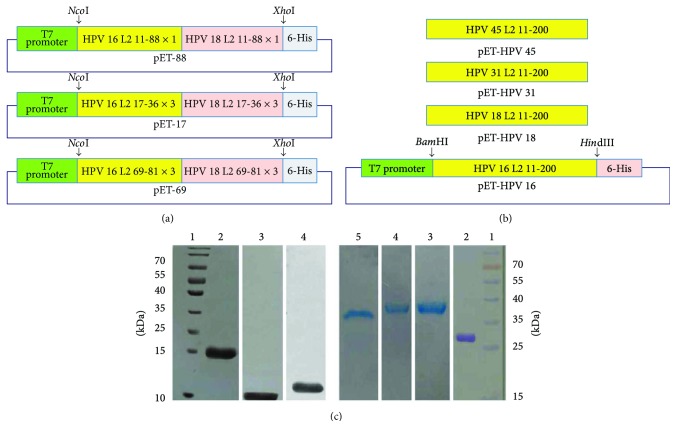
(a) Schematic diagram of the recombinant pET-28a plasmids encoding dual-type fusion peptides. (b) Schematic representation of recombinant pET-HPV 16, pET-HPV 18, pET-HPV 31, and pET-HPV 45 plasmids harboring L2 11-200 from HPV types 16, 18, 31, and 45, respectively. (c) Purification of L2 dual-type fusion peptides and L2 proteins amino acids 11-200. The expressed proteins were purified by Ni-NTA affinity chromatography and purified polypeptides were visualized in Coomassie-stained gels. Lanes in (a): 1: molecular weight marker; 2, 3, and 4: purified DT 11-88 × 1, 69-81 × 3, and 17-36 × 3 fusion peptides, respectively. Lanes in (b): 1: molecular weight marker; 2, 3, 4, and 5: purified L2 11-200 proteins from HPV 18, 16, 31, and 45, respectively.

Three “dual-type DNA sequences” were designed and synthesized for immunization studies. The DNA sequences encoded the three head-to-tail tandem repeats (multimers) of either HPV L2 RG-1 epitope “17-36 residues (17-36 × 3)” or “69-81 residues (69-81 × 3)” or one copy (monomer) of 11-88 amino acids (11-88 × 1) from HPV 16 (accession number: NC_001526) fused to the same regions, with the same patterns (i.e., 17-36 × 3 or 69-81 × 3 or 11-88 × 1, resp.) of HPV 18 (accession number: NC_001357). All DNA sequences were codon optimized for expression in *E. coli* host, synthesized (Biomatik, Canada) and subcloned into the 5′* Nco*I and 3′* Xho*I restriction sites of the pET-28a (+) expression vector (Addgene) in upstream of the C-terminal His-tag sequence. The three recombinant vectors corresponding to the abovementioned sequences are termed as the following: pET-28-HPV 17-36 × 3 (hereafter; pET-17), pET-28-HPV 69-81 × 3 (hereafter; pET-69), and pET-28-HPV 11-88 × 1 (hereafter; pET-88), respectively (Figure 1).

To assess the spectrum and potential of cross-reactive antibodies (IgGs) induced by immunization of the dual-type fusion L2 peptides via ELISA-based neutralization assays, the coding sequences of L2 amino acids 11-200 from HPV types 16, 18, 31, and 45 (accession numbers: NC_001526, NC_001357, J04353, and X 74479, resp.) were also codon optimized for expression in *E. coli* host, synthesized (Biomatik, Canada) and subcloned into the 5′* Bam*H I and 3′* Hin*dIII restriction sites of the pET-28a (+) expression vector (Addgene). The recombinant vectors corresponding to the abovementioned sequences, encoding the four HPV L2 amino acids 11-200 peptides, are termed as the following: pET-HPV 16, pET-HPV 18, pET-HPV 31, and pET-HPV 45 (Figure 1).

The final recombinant constructs were confirmed by restriction analyses followed by agarose gel electrophoresis and DNA sequencing reactions. All molecular and cloning procedures were performed according to standard protocols [37].

### 2.2. Expression, Purification, and Analyses of the HPV L2 Peptides

All three recombinant vectors encoding the dual-type fusion L2 peptides (pET-17, pET-69, and pET-88) and the four recombinant vectors encoding the L2 amino acids 11-200 from HPV types 16, 18, 31, and 45 (pET-HPV 16, pET-HPV 18, pET-HPV 31, and pET-HPV 45) were transformed into *E. coli* BL21 (Rosetta DE3) and induced for protein expression by IPTG induction (1 mM). The expressed proteins were further purified by Ni-NTA affinity chromatography according to the denaturing protocol of the manufacturer from the sonicated bacterial lysate (Qiagen, Germany). Subsequently, the recombinant polypeptides were renatured by dialysis against phosphate-buffered saline (PBS) and quantified using Bradford protein assay (Thermo Fisher Scientific, USA). The endotoxin level of the purified dual-type fusion peptides was further quantified by QCL-1000 chromogenic limulus amoebocyte lysate test (BioWhittaker) according to the manufacturer protocols. The recombinant protein was stored at −70°C until use.

Expression of all the peptides was analyzed by 12% SDS-PAGE according to standard protocols [38]. Specificity and integrity of the expressed peptides were determined by Western blot analysis using mouse anti-6 × His-HRP monoclonal antibody (Abcam, Cambridge, UK). Detailed method is described in supplementary dataset1.

### 2.3. Preparation of Immunogens and Immunization of Mice

All animal experiments were performed in accordance with the institutionally approved protocols of Pasteur Institute of Iran. Groups of five female Balb/c (H-2^d^) mice, 4–6 weeks age were used for immunization. In the first immunization experiments, to compare the potency of the different peptides to elicit neutralizing antibodies, separate mice groups were immunized subcutaneously (s.c.) with 25 *μ*g of each three dual-type fusion L2 peptides (in separate experiments), three times at two-week intervals. To this end, antigens were formulated in “complete Freund” adjuvant (CFA; Sigma) for the priming dose and in “incomplete Freund” adjuvant (IFA; Sigma) as booster immunizations in 100 *μ*l total immunogen volume (at 1 : 1 ratio) for each immunization (Table 1).

Second round of immunization experiments was performed to compare the potency of the different adjuvants to elicit neutralizing antibodies in formulation with the best identified antigen (by the results of the first round of immunization experiments) which was pET-88-encoding antigen; 11-88 × 1 HPV 16 + 11-88 × 1 HPV 18 (also called “dual-type 11-88 × 1 fusion peptide; DT: 11-88 × 1”). To this end, 25 *μ*g of the DT: 11-88 × 1 peptide was formulated in either aluminum hydroxide (alum; 50 *μ*g), or MF59 (at 1 : 1 ratio), or Montanide ISA 720 (M720; adjuvant/antigen ratio of 2 : 1), or Montanide ISA 50V (M50; at 1 : 1 ratio (in 100 *μ*l total immunogen volume for each immunization (Table 2). Immunization procedures were the same as mentioned above for first round of immunization experiments. Control groups were administered with 100 *μ*l of sterile PBS or CFA/IFA with similar procedures, respectively (Tables 1 and 2).

Blood samples were collected from all experimental groups through retroorbital bleeding two weeks after the final injection and sera were maintained in −20°C until use.

### 2.4. Production and Analyses of HPV 16 and 18 Pseudoviruses (PsVs)

To assess the neutralizing capacity of the immunized mice sera, we first generated the HPV 18 and HPV 16 pseudoviruses (PsV) as described elsewhere [39] with minor modifications. Briefly, 293 TT cells (2 × 10^7^) were cultured 20 hour before transfection in 162 cm^2^ flasks containing Dulbecco's modified Eagle's medium (high glucose) (Gibco, USA) supplemented with 10 U/ml penicillin, 10 *μ*g/ml streptomycin, and 10% FBS (Biosera, England). Cells were cotransfected with 40 *μ*g of a bicistronic L1 and L2 expression constructs (either HPV 16 L1/L2 (p16L1L2) or HPV 18 L1/L2 (p18L1L2); kindly provided by Professor Martin Muller, DKFZ, Germany) and 40 *μ*g of a GFP expression packaging plasmid (pfwB) (Addgene number 37329) in two different reactions (for HPV 16 L1/L2 and HPV 18 L1/L2) using Lipofectamine 2000 transfection reagent (Thermo Fisher Scientific, US). Media were replaced 6 h after transfection and cells were harvested 48 hours post transfection by centrifugation at 300*g* for 10 min and lysed by resuspension (108 cells/ml) in DBPS (Thermo Fisher Scientific) supplemented with 9.5 mM MgCl_2_, 0.5% Brij 58 (*w/v*; Sigma), and 0.2% Benzonase followed by overnight incubation at 37°C to induce pseudovirion maturation. The next day, crude extract of cell lysates was chilled and subjected to salt extraction (adjusted to 0.8 M NaCl) and further incubated at 37°C for 1 h. Subsequently, cell lysates were clarified by spinning at 1500 ×g for 10 min at 4°C. Finally, supernatants were harvested and stored at −80°C until use.

Generation of HPV 16 L1/L2 and HPV 18 L1/L2 PsVs (PsV 16 and PsV 18, resp.) was confirmed by transducing 293 TT cells using crude extract of cell lysates at 1 : 100 dilution in 24-well plates for each PsV and direct observation of GFP-expressing cells under fluorescence microscope (Motic AE31E Inverted Microscope, USA) 48 h after transduction. To measure the infectious titers of PsV, the 293 TT cells (1 × 105 cells/well) were transduced with serial dilutions of PsV preparations and 48 h post transduction, GFP-expressing cells were analysed by flow cytometry (PASIII, Partec, Germany). Finally, the infectious titers were calculated as previously described for lentiviral vector titration [40, 41] and reported by transduction unit (TU/ml).

For further visualization and confirmation of PsVs, extracted suspension (50 *μ*l) of abovementioned preparations was placed on a 400-mesh Formvar/carbon-coated copper grid and after absorption, the grids were washed briefly and stained with 2% phosphotungstic acid for 2 min. Subsequently, the stain was removed, and the grid was allowed to air dry for 30 minutes. The microscopy was performed with transmission electron microscopy (TEM) (Zeiss 10A). Micrographs of random sections were taken at a magnification of 50,000x.

### 2.5. Enzyme-Linked Immunosorbent Assays (ELISA)

An in-house-developed ELISA was designed to assess the titer of the cross-reactive antibody (IgG) of mice immunized by DT: peptides to HPV L2 11-200 peptides of types 16, 18, 31, and 45 (encoded by pET-HPV 16, pET-HPV 18, pET-HPV 31, and pET-HPV 45 vectors, resp.). To this end, ELISA 96-well plates (Nunc) were coated with 500 ng/well of recombinant HPV L2 amino acids 11-200 from HPV types 16, 18, 31, and 45 in separate wells (overnight) as described before [23]. The coated plates were blocked with 1% bovine serum albumin (BSA) at 4°C and incubated with tenfold serially diluted serum of immunized mice from each group separately for 1 h at RT. After washing with PBST (PBS 0.05% tween), 100 *μ*l of HRP-conjugated goat anti-mouse immunoglobulin G (Abcam, Cambridge, UK) (1 : 20000) was added to each well and incubated for 1 hour at RT. After extensive washing steps, reactions were developed by adding 100 *μ*l of 3,3′,5,5′-tetramethylbenzidine (TMB) substrate (Abcam, Cambridge, UK), and color development was stopped by 2N sulfuric acid. Finally, the optical density of wells was measured at 450 nm by ELISA reader (BioTek, USA). Antibody (IgG) titers were expressed as the reciprocal of the highest dilution at which the OD 450 was greater than 2-fold in comparison with the same dilution at control sera.

Antibody IgG subclasses and isotypes (IgG1, IgG2a, and IgG2b) in the sera of mice immunized with different adjuvants formulations of the L2 11-88 fusion peptide were measured as described above using 1 : 4000 and 1 : 2000 dilution of sera and HRP-labeled goat anti-mouse conjugates of IgG subtypes (Santa Cruz, USA), respectively.

In all experiments, the cut-off value was taken as twice the mean of absorbance values of the negative control (unimmunized mice sera) and each sample was tested in triplicate.

### 2.6. *In Vitro* Neutralization Assay

The generated HPV 18/16 PsVs were used for performing the neutralization assay as previously described [39] with few modifications. Briefly, 20,000 293 TT cells were seeded in Dulbecco's modified Eagle's medium (high glucose) (Gibco, USA) supplemented with 10 U/ml penicillin, 10 *μ*g/ml streptomycin, and 10% FBS (Biosera, England) in each well of a 96-well plates (SPL, South Korea). The day after, pooled sera from each immunized group (5 mice per group) were serially diluted (at starting dilution 1 : 10) in culture media. Subsequently, 20 *μ*l of diluted sera was mixed with 80 *μ*l of diluted PsV (1 : 100), incubated on ice for 1 h and added to the preplated 293 TT cells. Two days after infection, the cells were trypsinized and GFP-expressing cells were analyzed by flow cytometry. Cells treated with the only diluted PsV were considered as negative control. Neutralization titers were determined as the highest sera dilution of each immunized group at which the HPV 16 and HPV 18 PsVs were neutralized (at least 50%) in comparison with cells infected with the diluted PsV without sera.

### 2.7. Statistical Analysis

Experimental data were analyzed using GraphPad Prism 6.0 (GraphPad Software, San Diego, CA). Significant differences among experimental groups were determined using one-way analysis of variance (ANOVA) and Bonferroni multiple comparison test. Significant differences between groups were set at *P* values less than 0.05.

## 3. Results

### 3.1. Production of HPV L2 Peptides in *E. coli*

Restriction analyses of all seven recombinant pET-28a (+) plasmids (Supplementary Fig 1) and DNA sequencing results (data not shown) confirmed the proper construction of the expression vectors with the expected size of the inserted genes. Restriction analyses of the three constructs harboring the genes corresponding to the dual-type fusion L2 peptide by *Nco*I/*Xho*I restriction enzymes followed by agarose gel electrophoresis resulted in two bands of 5350 bp corresponding to the body of digested pET-28a vector and 385 bp, 300 bp, or 500 bp for the fragment inserted into pET-17, pET-69, and pET-88, respectively (supplementary Fig.  1). Accordingly, digestion and sequence analyses of all four pET-28-HPV L2 (11-200) vectors corresponding to types 16, 18, 31, and 45 resulted in two fragments of 5360 bp corresponding to the body of digested pET-28 vector and 600 bp corresponding to the fragment inserted into pET-HPV 16, pET-HPV 18, pET-HPV 31, and pET-HPV 45 (Supplementary Fig.  1).

Induction of *E. coli* BL21 (Rosetta DE3) harboring the dual-type fusion L2 peptide-encoding pET-28a (+) vectors by IPTG resulted in the expression of recombinant protein with molecular weight (MW) of ~14 kDa, 11 kDa, and 17 kDa corresponding to expressed peptides of pET-17, pET-69, and pET-88, respectively (Supplementary Fig.  2A). Accordingly, the observed size for the protein bands in Coomassie blue-stained SDS-PAGE was comparable to the calculated size of the DT: L2 peptides for a total of 120 amino acids (DT: 17-36 × 3; 17-36 × 3 HPV 16 + 17-36 × 3 HPV 18), 80 amino acids (DT: 69-81 × 3; 69-81 × 3 HPV 16 + 69-81 × 3 HPV 18), and 154 amino acids (DT: 11-88 × 1; 11-88 × 1 HPV 16 + 11-88 × 1 HPV 18) with addition of 6 × His-tag and flanking regions for each corresponding constructs. Similarly, the calculated size of the L2 11-200 peptide for a total of 190 amino acids might be around 25 kDa. Interestingly, however, induction of *E. coli* BL21 (Rosetta DE3) harboring the L2 11-200 peptides-encoding pET-28a (+) vectors by IPTG resulted in the expression of distinctive bands with various MVs of 38 kDa, 28 kDa, 38 kDa, and 36 kDa corresponding to expressed peptides from pET-HPV 16, pET-HPV 18, pET-HPV 31, and pET-HPV 45 vectors, respectively (Supplementary Fig.  2C). This interesting point will be further elucidated in Discussion. Western blot analyses confirmed the induction of the protein bands as the expected dual-type fusion L2 peptides and the L2 11-200 peptides (Supplementary Fig.  2B and 2D, resp.). Ni-NTA-based affinity chromatography purification of the proteins indicated homogenous bands for all expressed peptides corresponding to the observed sizes in SDS-PAGE analyses (Figure 1(c)). This final result indicated the stability and purification of the peptides. Quantification of the endotoxin levels indicated less than 25 endotoxin units per 50 *μ*g for all three purified dual-type fusion proteins, which were appropriate for the final aim of the immunization.

### 3.2. Characterization of the Generated PsV 16/18

**Figure 2:**
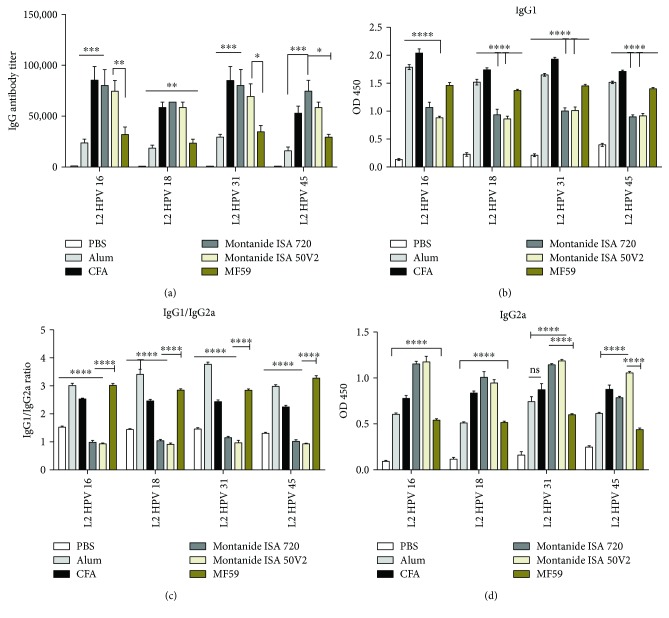
Cross-reactive antibody responses that elicited in mice immunized with the dual-type L2 11-88 × 1 peptide emulsified in different adjuvants. Mice immunized (s.c.) three times at two-week interval with 25 *μ*g of L2 11-88 × 1 formulated in alum (50 *μ*g), CFA (at 1 : 1 ratio), Montanide ISA 720 (adjuvant/antigen ratio of 2 : 1), Montanide 50V2 (at 1 : 1 ratio), and MF59 (at 1 : 1 ratio). Mice administered with PBS were regarded as negative control. Two weeks after the last vaccination, blood samples were collected, and after preparation of twofold serially diluted sera from 1 : 100, cross-reactive antibody (IgG) titers were detected by end point dilution ELISA (a). In addition, IgG isotypes IgG1 (b), IgG2a (c), and IgG1/IgG2a ratio (d) were measured at a dilution (1 : 4000). Data are represented as means ± standard error of means (SEM) of triplicate from 5 mice per group. ^∗∗∗∗^ *P* < 0.0001, ^∗∗∗^ *P* < 0.001, ^∗∗^ *P* < 0.01, and ^∗^ *P* < 0.05.

Generation of pseudovirions was initially confirmed by direct observation of GFP-expressing 293 TT cells under fluorescence microscope 48 h after transduction. As shown in Figure 2(c), a great percentage of the cells transduced by crude extract of cell lysates from either PsV 16 or PsV 18 exhibited GFP expression. Accordingly, flow cytometry analyses indicated that around 25% and 20% of the cells transduced by PsV 16 or PsV 18, respectively, were GFP positive (Figures 2(a) and 2(b)) and calculation of the infectious titers based on these titers (as described in Materials and Methods) indicated a titer of 5 × 10^6^ IU/ml and 4 × 10^6^ IU/ml for PsV 16 and PsV 18 crude stocks, respectively. Finally, as shown in Figure 2(d), direct visualization by electron micrographs of the generated PsVs indicated a polymorphic form with approximate size of 30–60 nm for both PsVs 16 and 18 (only results for PsV 16 is shown).

### 3.3. Cross-Reactivity and Neutralizing Capacity of the Antibodies Induced by Different Dual-Type Peptides

**Figure 3:**
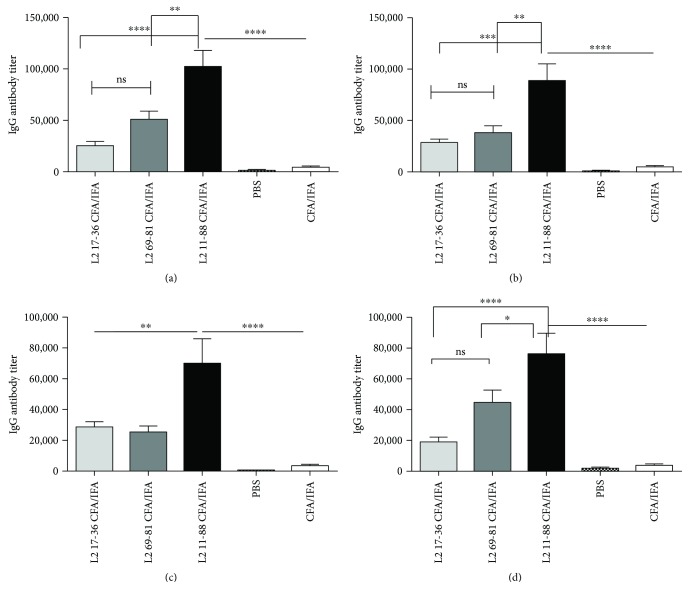
Cross-reactive antibody responses of three dual-type fusion peptides against protein L2 11-200 from various HPV types. Mice were immunized 3 times at two-week interval with dual-type peptides emulsified in CFA/IFA adjuvants. Two weeks after the last vaccination, blood samples were collected, and twofold serially diluted sera were prepared (starting dilution at 1 : 100) and antibody cross-reactive titers against HPV 16 L2 11-200 (a), HPV 18 (b), HPV 31 (c), and HPV 45 (d) were determined by end point dilution ELISA. Data are represented as means ± standard error of means (SEM) of triplicates from 5 mice per group. ^∗∗∗∗^ *P* < 0.0001, ^∗∗∗^ *P* < 0.001, ^∗∗^ *P* < 0.01, and ^∗^ *P* < 0.05. ns: not significant.

To assess the cross-reaction titers of the antibodies in mice immunized by CFA/IFA formulated, dual-type fusion L2 peptides (Table 1), the IgG levels were evaluated via ELISA against recombinant HPV L2 11-200 peptides (encoded by pET-HPV 16, pET-HPV 18, pET-HPV 31, and pET-HPV 45 vectors) in four separate assays. As shown in Figure 3(a), sera of mice immunized with DT 11-88 × 1, 17-36 × 3, and 69-81 × 3 were able to react against HPV 16 with significant mean titers of 1 × 10^5^, 25 × 10^3^, and 5 × 10^4^, respectively, compared to PBS control group (titer < 50). Likewise, mice received the same mentioned peptides could elicit significant mean titers of 8 × 10^4^, 3 × 10^4^, and 26 × 10^3^ against HPV 18 (Figure 3(b)). Furthermore, mice administered with the same above peptides induce significant but lower cross-reactive antibody titers of 7 × 10^4^, 28 × 10^3^, and 22 × 10^3^ against HPV 31 (Figure 3(c)) and also titers of 74 × 10^3^, 2 × 10^4^, and 42 × 10^3^ against HPV 45 (Figure 3(d)), respectively. While both DT: 17-36 × 3 and DT: 69-81 × 3 peptides showed almost similar titers in each HPV type cross-reaction ELISA (with no significant differences), the DT: 11-88 × 1 peptide demonstrated the highest cross-reactive titers in all four assays (Figure 3).

**Figure 4:**
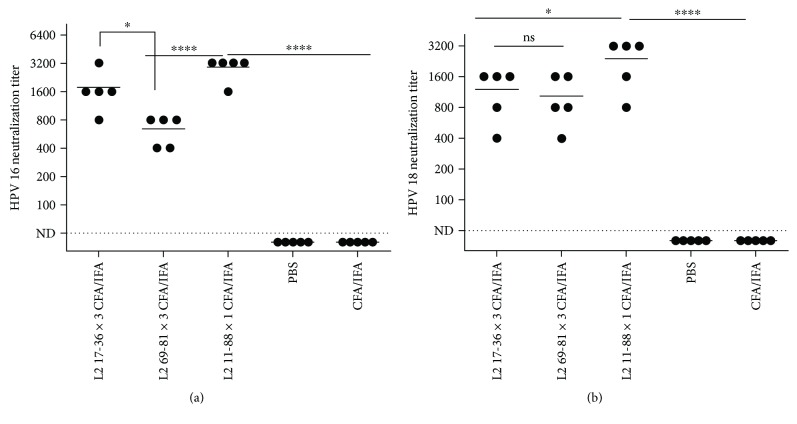
Neutralizing antibody responses in mice vaccinated with dual-type fusion peptides. Mice (5/group) were immunized (s.c.) three times at two-week interval with either the dual-type fusion peptides L2 17-36 × 3, 69-81 × 3, and 11-88 × 1 emulsified in CFA/IFA. Two weeks after the last vaccination, blood samples were collected and twofold serially diluted sera (starting dilution at 1 : 50) were tested for *in vitro* neutralization of either HPV 16 (a) or HPV 18 (b) pseudovirions. The neutralizing antibody titers were expressed as the reciprocal dilution at which 50% of the pseudovirions were neutralized compared to positive control wells. End point titers were plotted and means reflected as horizontal lines. A neutralizing titer of <1 : 50 was considered as not detectable (ND). ^∗^ *P* < 0.05 and ^∗∗∗∗^ *P* < 0.0001. ns: not significant.

Similarly, evaluation of the induced antibodies for neutralization of PsV 16/PsV 18 indicated that the DT: 11-88 × 1 demonstrated the cross-neutralizing antibody mean titers of 3150 and 2400 against PsV 16 (Figure 4(a)) and PsV 18 (Figure 4(b)), respectively. Both DT: 17-36 × 3 and DT: 69-81 × 3 peptides showed almost similar cross-neutralizing titers towards PsV 18, but in case of PsV 16, cross-neutralizing antibody titers were significantly higher (*P* < 0.05) for DT: 17-36 × 3 peptide (Figures 4(a) and 4(b)). The comparative “*P* values” for each antibody titer are provided in Figures 3 and 4. No cross-reactivity was observed for sera of mice immunized by PBS or CFA/IFA alone (Figure 4).

### 3.4. Cross-Reactivity and Neutralizing Capacity of the Antibodies Induced by DT: 11-88 × 1 Peptide Formulated in Different Adjuvants

Having shown that immunization by the DT: 11-88 × 1 peptide induced the highest cross-reactive antibody titers among the three studied dual-type peptides, we assessed the best adjuvant formulation for this peptide (Figure 2). Evaluation of the different adjuvant formulations of the 11-88 peptide by ELISA for the spectrum of the cross-reactivity indicated that all used adjuvants, similar to CFA/IFA, cross-reacted with all four types of HPV L2 11-200 peptides (types 16, 18, 31, and 45), albeit in lesser titers. Similarly, the level of antibody titers for all adjuvant formulations also followed the same pattern for different HPV types, i.e., highest against type 16 followed by types 18, 45, and 31. Differences between groups and corresponding *P* values are indicated in Figure 2(a). Among utilized adjuvants, alum induced the least titers (mean titer in all groups: 22 × 10^3^), followed by MF59 (mean titer in all groups: 31 × 10^3^), while both Montanide adjuvants (ISA 720 and 50; mean titers were 7 × 10^4^ and 64 × 10^3^, resp.) elicited the highest and almost similar titers comparable to that of CFA/IFA (mean titer: 68 × 10^3^) (Figure 2(a)).

Results of IgG subtyping for different adjuvant formulations of the DT: 11-88 × 1 peptide indicated that in all groups (except in Montanide ISA 720 and 50 for all HPV types), IgG1 (Figure 2(b)) was almost the predominant isotype (Th2-biased response) followed by IgG2a (Figure 2(d)). Accordingly, for all these groups, IgG1/IgG2a ratios (Figure 2(c)) of serum were higher than 1.0, suggesting a tendency for switching to Th2 polarization of the immune responses. However, induction of comparable titers of IgG1 and IgG2a in case of Montanide adjuvants (M720 and 50) might be an indication of switching towards both Th1 and Th2 immune responses.

**Figure 5:**
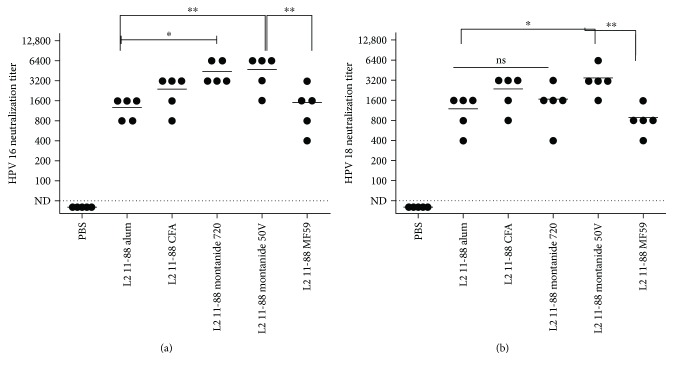
Neutralizing antibody responses in mice vaccinated with the dual-type L2 11-88 × 1 peptide formulated in different adjuvants. Mice (5/group) were immunized (s.c.) three times at two-week interval with 25 *μ*g of L2 11-88 × 1 formulated in alum (50 *μ*g), CFA (at 1 : 1 ratio), Montanide ISA 720 (adjuvant/antigen ratio of 2 : 1), Montanide 50V2 (at 1 : 1 ratio), and MF59 (at 1 : 1 ratio). Mice administered with PBS were regarded as negative control. Two weeks after the last vaccination, blood samples were collected and twofold serially diluted sera (starting dilution at 1 : 50) were tested for *in vitro* neutralization of either HPV 16 (a) or HPV 18 (b) pseudovirions. The neutralizing antibody titers were expressed as the reciprocal dilution at which 50% of the pseudovirions were neutralized compared to positive control wells. End point titers were plotted and means reflected as horizontal lines. A neutralizing titer of <1 : 50 was considered as not detectable (ND). ^∗^ *P* < 0.05 and ^∗∗^ *P* < 0.01. ns: not significant.

In accordance with cross-reactivity results, evaluation of the induced antibodies by different adjuvant formulations of the DT: 11-88 × 1 peptide for neutralization of PsV 16/PsV 18 also indicated the higher neutralization titers for PsV 16 (mean titer of 50: 4800 and ISA 720: 4600) compared to PsV 18 (mean titer of 50: 3800 and ISA 720: 1600) which was highest for ISA 50 and 720 (almost similar) followed by CFA (mean titer: 2400 for PsV 16 and 2800 for PsV 18), MF59 (mean titers for PsV 16 and PsV 18 were 1600 and 800, resp.), and alum (mean titers for PsV 16 and PsV 18 were 1400 and 1200, resp.), respectively (Figures 5(a) and 5(b)). The comparative “*P* values” for each antibody titer are provided in Figures 2 and 5. No cross-reactivity was observed for sera of mice immunized by PBS or adjuvants alone (Figure 2(a)).

## 4. Discussion

In the present study, we demonstrated the stable expression and proper purification of three dual-type fusion HPV L2 peptides in *E. coli* host. These three peptides represented the most recognized immunogenic and conserved epitopes in the N-terminal of L2 peptide and encoded for three head-to-tail tandem repeats (multimers) of either HPV L2 RG-1 epitope “17-36 residues (17-36 × 3)” or “69-81 residues (69-81 × 3)” or one copy (monomer) of 11-88 amino acids (11-88 × 1) from HPV 16 fused to the same regions with the same patterns (i.e., 17-36 × 3 or 69-81 × 3 or 11-88 × 1, resp.) of HPV 18. Immunization studies in mice indicated that L2 11-88 peptide formulated in the human compatible adjuvant “Montanide ISA 720” could induce high levels of cross-reactive antibodies towards HPV types 16, 18, 31, and 45 and neutralization potencies to HPV 16 and HPV 18 PsVs.

Restriction analyses and agarose gel electrophoresis of the recombinant pET-28a (+) plasmids harboring the genes corresponding to the three DT L2 peptides resulted in the inserts, which in accordance with prior reports, were around 385 bp for of pET-36 (17-36 × 3 HPV 16 + 17-36 × 3 HPV 18) [20, 24], 300 bp for pET-69 (69-81 × 3 HPV 16 + 69-81 × 3 HPV 18), and 500 bp for pET-88 (11-88 × 1HPV 16 + 11-88 × 1 HPV 18) (Supplementary Fig.  1A). Similarly, results of these analyses for the pET-28-HPV L2 (11-200) vectors (encoding types 16, 18, 31, and 45) indicated 600 bp (Supplementary Fig.  1B) inserted fragments which were consistent with our expectation. Accordingly and in agreement with previous reports on expression of monomeric or various multimeric forms of these epitopes, SDS-PAGE and Western blotting analyses also indicated the proper expression of the dual-type fusion L2 peptides (Supplementary Fig.  2A and 2B) with expected MWs of approximately: 14 kDa for pET-36 encoded 17-36 peptide [20, 42], 11 kDa for pET-69 encoded 69-81 peptide, and 17 kDa for pET-88 encoded 11-88 peptide [20]. These results indicated that bacterial expression for the dual-type fusion L2 peptides was quite efficient and stable which is a very positive point compared to frequent reports on unstable expression of longer concatenated multimeric peptides corresponding to these epitopes [31, 43]. In fact, presence of degradation products has been reported as one of the main drawbacks for induction of strong immune responses and NAbs by long multimers of RG1 and 11-88 peptides [20, 29, 31, 32]. Interestingly, however, induction of *E. coli* cells harboring the L2 11-200 peptides-encoding pET-28a (+) vectors by IPTG resulted in the expression of distinctive bands with various MVs of 38 kDa, 28 kDa, 38 kDa, and 36 kDa corresponding to expressed peptides from pET-HPV 16, pET-HPV 18, pET-HPV 31, and pET-HPV 45 vectors, respectively (Supplementary Fig.  2C and 2D). This observation was in accordance with a prior report on variation of the size of HPV L2 peptides of different types despite similar number of amino acids have been already reported [44, 45]. The reason for this phenomenon is not fully understood as there are no known posttranslational modifications of L2 [45].

**Figure 6:**
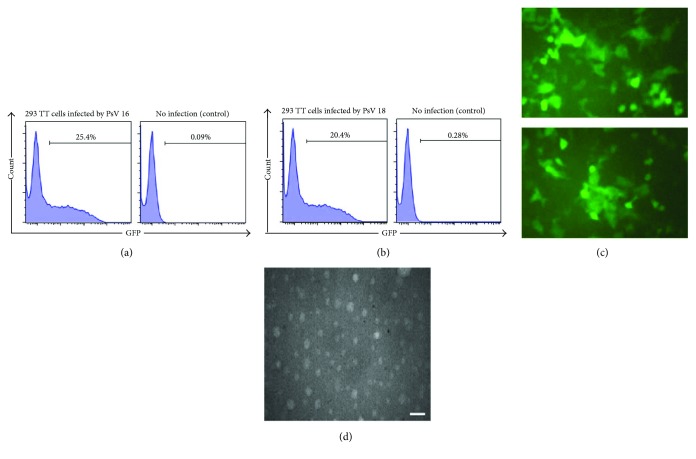
Generation and characterization of HPV 16 and HPV 18 pseudovirions. Two hundred ninety-three TT cells (2 × 10^7^) were cultured in 162 cm^2^ flasks. Twenty hours after culture, the cells were transfected with 40 *μ*g of either p16L1L2 or p18L1L2 plasmids and 40 *μ*g of the GFP-expressing reporter plasmid (pfwB). Generation of pseudovirions was confirmed by transducing 293 TT cells using crude extract cell lysates at a 1 : 100 dilution in 24-well plates in triplicate for each PsV. Forty-eight hours after infection, cells expressing GFP were analyzed by flow cytometry (a and b) and observed under inverted microscope (c). Furthermore, crude cell extract lysates containing PsV 16 were also subjected to transmission electron microscopy (visualized at a magnification of 50,000) to confirm particles (d).

Microscopic and flow cytometry analyses (Figure 6) indicated the efficient production of HPV 16/18 PsVs with proper size and infectious titers for performing the neutralization assays [46, 47]. Application of the HPV PsV technology with a reporter gene within the papillomavirus L1 and L2 capsids (which has been made in the present study) is shown to be determinant in accurate measurement of neutralizing antibodies and mouse challenge models [48, 49]. Results of the ELISA (Figure 3) and neutralization assay (Figure 4) for the NAbs induced via immunization by three DT L2 peptides provided five main conclusions: (i) Abs could cross-react with HPV types 16, 18, 31, and 45, (ii) both cross-reactions and neutralization capabilities of NAbs were better for type 16 compared to 18, (iii) Ab cross-reactions were much higher for the endogenous vaccine-derived epitopes (HPV 16/18) compared to heterologous (HPV 31/45) types, (iv) Ab cross-reactions were better for type 45 compared to 31, and (v) both cross-reactions and neutralization capabilities of Abs were much higher for DT: 11-88 × 1 (*P* < 0.05) than the other two peptides, but almost similar for both DT: 17-36 × 3 and DT: 69-81 × 3 peptides (*P* > 0.05). Although, to our knowledge, no prior study has addressed comparing of any monomeric/multimeric forms of all these three peptides together for immunization studies, but some reports on comparing either of the two pair of the peptides have provided results which are consistent with our conclusions. In an excellent study by Jagu et al. [20], immunization studies in mice and rabbits by a 49 kDa concatenated RG1-encoding peptides of 22 clinically relevant HPV genotypes (i.e., 17-36 × 22) and a 43 kDa peptide corresponding to 11-88 × 5 peptide have shown similar results to ours. Both immunogens in this prior study (similar to that of ours) included HPV types 16 and 18, while 17-36 × 22 peptide also included that of HPV 31 and 45. However, 11-88 × 5 induced remarkably higher titers of NAbs towards HPV types 16 and 18 (vaccine-derived epitopes) and even 31 and 45 (not included in the vaccine) compared to that of 17-36 × 22 peptide [20]. In a confirming recent study, the same investigators [29] demonstrated that a fusion peptide of divergent HPV L2 11-88 × 8 peptides (HPV 6, 16, 18, 31, 39, 51, 56, and 73) induced significantly higher NAbs than that of 13-47 × 15 peptides (HPV 6, HPV 11, HPV 16, HPV 18, HPV 31, HPV 33, HPV 35, HPV 39, HPV 45, HPV 51, HPV 52, HPV 56, HPV 58, HPV 59, and HPV 73). Results of this prior study also indicated that the passive transfer of mice with rabbit antisera to HPV 16 17-36 and 65-81 peptides provided significant and similar protection rates against HPV 16 challenge which is in agreement with identical cross-reactivity results for these two peptides in our study (Figure 3). It was suggested that the reason for the less effectiveness of 17-36 than 11-88 multimeric fusion peptides might be the result of weak CD4 T cell help [50], which might be considered as the reason for the higher titers of NAbs obtained by DT: 11-88 × 1 in our study compared to that of the DT: 17-36 × 3 and DT: 69-81 × 3 peptides, too (Figures 4(a) and 4(b)). It should be noted that the ability of these multimeric L2 peptides to elicit immune response to HPV types not present in the immunogen (although in lower titers) may reflect the cross-linking and activation of B cells that recognize L2-specific neutralizing epitopes common to multiple HPV types in the fusion constructs [51, 52]. In accordance with our results (Figure 4), Jagu et al [20] also reported higher NAbs raised against type 16 compared to type 18. Rise of the higher titers of HPV 16 neutralization Abs compared to that of type 18 was also reported in the natural course of infection and might indicate the superior immunogenicity of this HPV type compared to others [53]. Of note however, although HPV 31 is more closely related to HPV 16 than HPV 45 which is more closely related to HPV 18 [6], but our results in agreement with this prior study also indicated higher levels of NAbs against type 45 compared to that of 31, for both 11-88 and 13-37 peptides. In fact, consistent with our result, several prior studies on immunization studies by monomeric/multimeric forms of HPV 16 RG1 epitope [17–27, 29, 31–35, 37–39] have also reported lower NAb titers for HPV type 31 [26, 33, 43, 50].

Adjuvants have a crucial role in subunit vaccine formulations to generate and enhance the weak immune responses induced by peptide antigens. Of note, antibody levels induced by L2 peptides are very weak compared to those induced by L1 VLP vaccines. Therefore, adjuvants have a critical role in formulation of L2 peptides as subunit vaccines to induce robust antibody responses. However, currently approved adjuvants for human use, like aluminum compounds, induce relatively weak responses and often require multiple immunizations to elicit protective antibody levels [28]. In this context, formulation of HPV L2 peptides with various adjuvants was reported and their potency for induction of neutralizing antibodies has been compared [19]. Although, the adjuvants evaluated in the present study (alum, MF59, and M720 and 50) are among the most appreciated ones for HPV L2 peptides formulation and immunization, but to our knowledge, no prior report has addressed their comparison for formulation of these antigens in a single study. As shown in Figure 2(a), evaluation of different adjuvant formulations of the dual-type 11-88 peptide (DT: 11-88 × 1) by ELISA indicated that alum induced the least titers, followed by MF59, while both Montanide adjuvants (ISA 720 and 50) elicited the highest and almost similar titers comparable to that of CFA/IFA. The same pattern was also followed by neutralization assays on PsV 16/PsV 18 (i.e., alum < MF59 < M720 and M50; Figures 2(a) and 2(b)). Alum is a particulate adjuvant and the most used adjuvant in HPV L2 immunization studies. Alum creates a depot for slow releasing of antigen and its adjuvant properties might be directly related to the biochemical properties of the antigen as well as its resulting adsorption capacity, which might either enhance or reduce antibody responses [54, 55]. As shown in Figure 2(a), immunization by DT: 11-88 × 1 + alum raised sufficient but lower titers of antibodies compared to other formulations. Our results are consistent with prior reports on immunization by HPV L2 11-200 × 3 + alum for raise of sufficient Abs [20]. However, in further agreement with more recent reports on immunization by HPV L2 11-88 × 3 + alum [29] and HPV L2 17-36 peptide displayed on adeno-associated virus (AAV) + alum [42], our results also indicated to the need for further enhancement of immune responses by addition of extra compounds like monophosphoryl lipid A (MPL), CPG, or RIBI (MPL + TDM) to the alum [29, 42]. Indeed, combination of alum + MPL found the most preferred combination for formulation of HPV L2 peptides and several immunization studies reported its application, including HPV L2 20-38 × 3 (incorporating from HPV 16, HPV 31, and HPV 51) fused with bacterial thioredoxin (Trx) protein that induced pan-type neutralizing and protective Abs in mice and guinea pigs [56]; HPV 16 L1-VLPs displaying the RG1 epitope (HPV L2 17-36 peptide) that induced broadest spectrum of cross-neutralizing antibodies in mice and rabbits [26]; recombinant adenovirus type 5 (Ad5) displaying HPV 16 L2 12-41 peptide which only protected mice from homologous (HPV 16) challenge [57]; and a fusion protein containing HPV 16 RG1 × 3 (E3) and a modified human IgG1 Fc scaffold (R4) in tandem (E3R4) that elicited cross-neutralizing Abs against HPV types 16, 18, and 6 in rabbits only when formulated with the alum + MPL combination [43]. Of note, in this final study, E3R4 formulated in Freund or MPL induced different levels of cross-neutralizing antibodies, indicating to the contributing role of the animal under study (besides adjuvant, used antigen or the vaccine scaffold) for induction of the neutralizing antibodies.

MF59 (Novartis), an oil-in-water adjuvant, was used in the first clinical trial of seasonal influenza virus vaccine around a quarter of century ago (in 1992) and is now licensed worldwide in 30 countries and has been used in a number of subunit vaccine formulation studies [58]. As shown in Figure 2(a), immunization by DT: 11-88 × 1 + MF59 raised sufficient but lower titers of antibodies compared to other formulations, except that of alum. To our knowledge, there is no prior report on immunization of this peptide with MF59. However, our results are somehow consistent with a recent report on immunization by an *E. coli*-derived, lipidated triple-repeat HPV 16RG-1 epitope fused with a FcgRI-specific single-chain antibody fragment (H22scFv) formulated with MF59 and poly I:C for raise of sufficient Abs [31]. Results of this prior study indicated to the absolute need of the used adjuvant (MF59 and poly I:C) for induction of sufficient titers of NAbs. Although no data on formulation of either adjuvant component alone (MF59 or Ploy I:C) is provided in the study, need for further enhancement of the immune responses might be the reason for this combination of two adjuvants.

The Montanide (M720 and 50) adjuvants are relatively safe and have been studied in several therapeutic and prophylactic vaccine studies contain natural metabolized oils and a highly refined mannide that acts as a depot to slow the release of Ag. These adjuvants have been also targets of studies for formulation of cancer vaccines and improving Ag presentation to T cells. However, only M720 is known to be human compatible [59, 60]. As shown in Figure 2(a), our results indicated that both Montanide adjuvants (ISA 720 and 50) elicited the highest and almost similar titers comparable to that of CFA/IFA. These results are in accordance with a recent report on immunization studies in mice by a double inserted/displayed RG-1 epitope (from HPVs 16 and 31) on adeno-associated virus 2 (AAV2) [27]. Induction of higher titers of cross-NAbs against HPV 16/18/31/45/52/58 was shown for application of the Montanide ISA 51 rather than MPL for this immunogen. Similarly, formulation of various multimeric forms of *E. coli*-derived HPV 16 L2 20-38 peptide with M720 adjuvant was also shown to be highly effective in induction of cross-NAbs against HPV types 16/33/58 [32].

Although, the contribution of cellular responses might not be completely neglected, the crucial role of neutralizing antibodies to confer sterilizing immunity against HPV infection is well documented [61]. Thus, induction of Th2-biased humeral responses by vaccination against this infection might be preferred. Cytokines such as IFN-*ϒ* and IL-4 (which are associated with IgG2a and IgG1 responses, resp.) are known to be secreted by Th1 and Th2 cells, respectively [62]. Accordingly, we performed IgG subtyping to gain some insights on the tendency of the different adjuvant formulations of 11-88 peptide for T-helper switching (Th1 versus Th2), although to our knowledge, no prior report has addressed this issue for HPV L2 peptides, to date. Results indicated IgG1/IgG2a ratios of higher than 1.0 (i.e., higher values of IgG1) for most groups (Figure 2(c)), suggesting a tendency for switching to Th2 polarization of the immune responses, while comparable titers of IgG1 and IgG2a in case of Montanide adjuvants (M720 and 50) indicating a relatively balanced Th1/Th2 immune responses. Both alum and MF59 are known as Th2-biased adjuvants capable of inducing high titers of IgG1 antibodies in mice and humans, which are associated with enhanced effect on generation of humeral responses [63–66]. These prior reports for alum and MF59 are in accordance with our results for induction of Th2-biased responses towards L2 11-88 peptide (Figure 2(b)). However, depending on the vaccination route or antigen type, aluminum hydroxide-based adjuvants might elicit both Th1/Th2 cellular responses [67], while MF59 might induce Th1 responses, too [59]. Consistent with our immunization results for M720 and 50 adjuvants, several prior studies indicated induction of strong and balanced Th1/Th2 immune responses using different antigen formulations such as hepatitis C virus proteins [68], subunit candidate vaccines against malaria [69], Leishmania major stress-inducible protein 1 [70], and *Schistosoma mansoni* cathepsin B [71]. The relevance of subclass antibodies against HPV L2 for the development of a protective immune response remains to be established. However, although directly not measured in this study, induction of IgG2a (Figure 2(d)) by these immunogens might be a demonstration of active helper T lymphocyte expansions through targeting CD4 + T cells, which are crucial for development of memory cells and long-term protection against infection [72].

Finally, it is noteworthy that although we did not compare our DT immunogens with currently available HPV L1-based vaccines (like Gardasil) or did not evaluate the cross-reactivity towards other important HPV types like HPV 58, but we consider the results of the prior studies [19, 20, 29]. We might assume similar observations. In this context, it might be expected that the sera from those receiving DT 11-88 × 1 formulated in M720 might induce HPV 16 and HPV 18 titers comparable to that of Gardasil, while the later might induce no (or only low and just detectable) levels of NAbs against HPV types 45 and 31. It might be also possible to further increase the titer of NAbs by this formulation through addition of other human compatible adjuvants like CpG to M720 as previously reported for a candidate hepatitis C virus vaccine in preclinical phase [72]. Accordingly, it might be expected that immunization by DT 11-88 × 1 formulated in M720 might also induce cross-reactive NAbs towards other important HPV types like HPV 58.

Taken together, to the best of our knowledge, this study presented the first report on construction of dual-type fusion L2 peptides (HPV 16/18) corresponding to the fused triplet epitopes of 17-36, 69-81, and 11-88 monomers and their proper/stable expression in bacterial system and comparing their potency for induction of NAbs. Our results indicated that all the three *E. coli*-derived DT peptides could raise efficient NAbs in mice, capable of cross-reacting with HPV types 16/18/31/45 and neutralizing HPV 16/18 PsVs, albeit with remarkably higher titers for DT: 11-88 × 1 peptide formulated in human compatible M720 adjuvant. Our results imply the possibility of development of a simple, low-cost preventive HPV vaccine based on this dual-type fusion L2 peptide with broad spectrum (at least effective against types 16, 18, 31, and 45 that are responsible for 90% of cervical carcinomas) for low-income countries.

## Figures and Tables

**Table 1:** Group of mice immunized with different dual-type L2 peptides formulated in CFA/IFA.

Groups	Immunogen
G1	(HPV 16) 17-36 × 3 + (HPV 18) 17-36 × 3 in CFA/IFA
G2	(HPV 16) 69-81 × 3 + (HPV 18) 69-81 × 3 in CFA/IFA
G3	(HPV 16) 11-88 × 1 + (HPV 18) 11-88 × 1 in CFA/IFA
G4 (control group)	CFA/IFA
G5 (control group)	PBS

CFA and IFA denote the complete and incomplete Freund adjuvants, respectively. CFA was used for formulation of the immunogen in first immunization and IFA for next immunizations. Control groups are deprived from antigen.

**Table 2:** Group of mice immunized with the dual-type 11-88 L2 peptide in different adjuvant formulations.

Groups	Immunogen^∗^
G1	(HPV 16) 11-88 × 1 + (HPV 18) 11-88 × 1 in alum
G2	(HPV 16) 11-88 × 1 + (HPV 18) 11-88 × 1 in CFA/IFA
G3	(HPV 16) 11-88 × 1 + (HPV 18) 11-88 × 1 in MF59
G4	(HPV 16) 11-88 × 1 + (HPV 18) 11-88 × 1 in M720
G5	(HPV 16) 11-88 × 1 + (HPV 18) 11-88 × 1 in M50
G6 (control groups)	Adjuvants (alum, MF59, M720, and M50)
G7 (control group)	PBS

^∗^CFA/IFA, alum, MF59, and M720 and M50 denote the complete/incomplete Freund, aluminum hydroxide, MF59, and Motanide ISA 720 and ISA 50V adjuvants, respectively. Control groups are deprived from antigen.

## Supplementary Materials

Supplementary Materials There have been some extra descriptions regarding the materials and methods that are removed in this final file of the manuscript. These sections are briefly include “A Detailed Description about Western Blot Analysis of the Expressed Peptides”, “Characterization of Recombinant Plasmids Encoding the Dual-Typed Fusion Peptides and L2 Amino Acids 11-200 from HPV types 16, 18, 31, and 45”, and “SDS-PAGE and Western Blotting of the Expressed L2 Dual-Typed Fusion Peptides and L2 Proteins Amino Acids 11-200”. It is noteworthy that a complete description regarding the removed sections is provided in Supplementary Materials file.
