# Supplementary material for: A Dual-Type L2 11-88 Peptide from HPV Types 16/18 Formulated in Montanide ISA 720 Induced Strong and Balanced Th1/Th2 Immune Responses, Associated with High Titers of Broad Spectrum Cross-Reactive Antibodies in Vaccinated Mice
Source: J Immunol Res. 2018 May 3;2018:9464186. doi: 10.1155/2018/9464186 (PMC5960516; doi:10.1155/2018/9464186)
Supplement: Supplementary Materials — There have been some extra descriptions regarding the materials and methods that are removed in this final file of the manuscript. These sections are briefly include “A Detailed Description about Western Blot Analysis of the Expressed Peptides”, “Characterization of Recombinant Plasmids Encoding the Dual-Typed Fusion Peptides and L2 Amino Acids 11-200 from HPV types 16, 18, 31, and 45”, and “SDS-PAGE and Western Blotting of the Expressed L2 Dual-Typed Fusion Peptides and L2 Proteins Amino Acids 11-200”. It is noteworthy that a complete description regarding the removed sections is provided in Supplementary Materials file. [file 9464186.f1.docx]

**Dataset1**

Western blot analysis was carried out by transferring the protein bands from polyacrylamide gel to nitrocellulose membrane via semi-dry blotting apparatus (Biorad, USA). Membranes were blocked with 5% blocking buffer over-night at 4°C. After several washing steps, the membrane was incubated with mouse anti-6×His-HRP monoclonal antibody (Abcam, Cambridge, UK) for 60 min at room temperature. Following washing steps, the membrane was incubated with HRP-conjugated anti-mouse IgG (Abcam, Cambridge, UK. Color visualization of the antigen-antibody reaction was accomplished using 3, 3’-diaminobenzidine tetra-hydrochloride solution (DAB) (Abcam, Cambridge, UK).

**Fig 1**


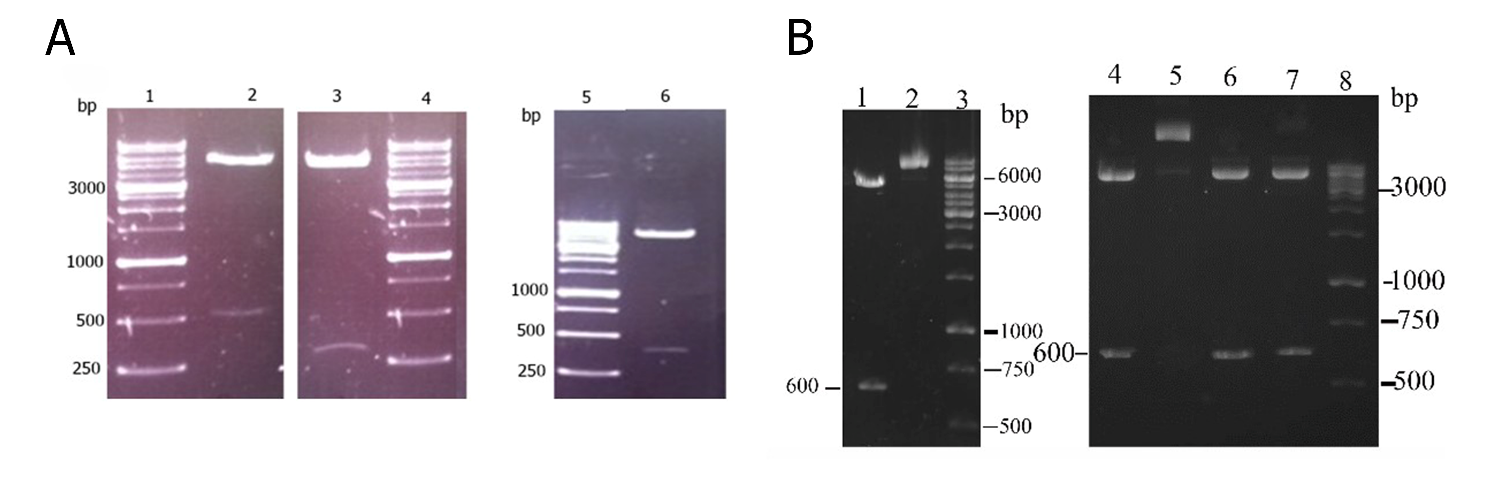


**Fig 1**. Characterization of recombinant plasmids encoding the dual-typed fusion peptides and L2 amino acids 11-200 from HPV types 16, 18, 31, and 45. (A) Agarose gel electrophoresis of digested recombinant plasmids by *Nco*Ι and *Xho* Ι restriction enzymes resulted in two fragments of 5350 bp, corresponding to the body of digested pET-28a vector, and 385 bp (lane 6), 300 bp (lane 3) or 500 bp (lane 2) fragment for fragments inserted into pET-17, pET-69 and pET-88, respectively. Lane 2: pET-88 (encoding 11-88 ×1), lane 3: pET-69 (encoding 69-81 ×3), lane 6: pET-17 (encoding 17-36 ×3). Lanes 1, 4 and 5: DNA marker. (B) Agarose gel electrophoresis of digested recombinant plasmids by *BamH*I and *Hind* IIΙ restriction enzymes resulted in two fragments of 5360 bp corresponding to the body of digested pET-28 vector and 600 bp for fragments inserted into pET-HPV16, pET-HPV18, pET-HPV31 and pET-HPV45. Lane 1: Digested pET-HPV16, Lane 2: Undigested pET-HPV16, Lane 4: Digested pET-HPV18, Lane 5: Undigested pET-HPV18. Lanes 6 and 7: Digested pET-HPV31 and pET-HPV45, respectively. Lane 3 and 8: DNA marker.

**Fig 2**


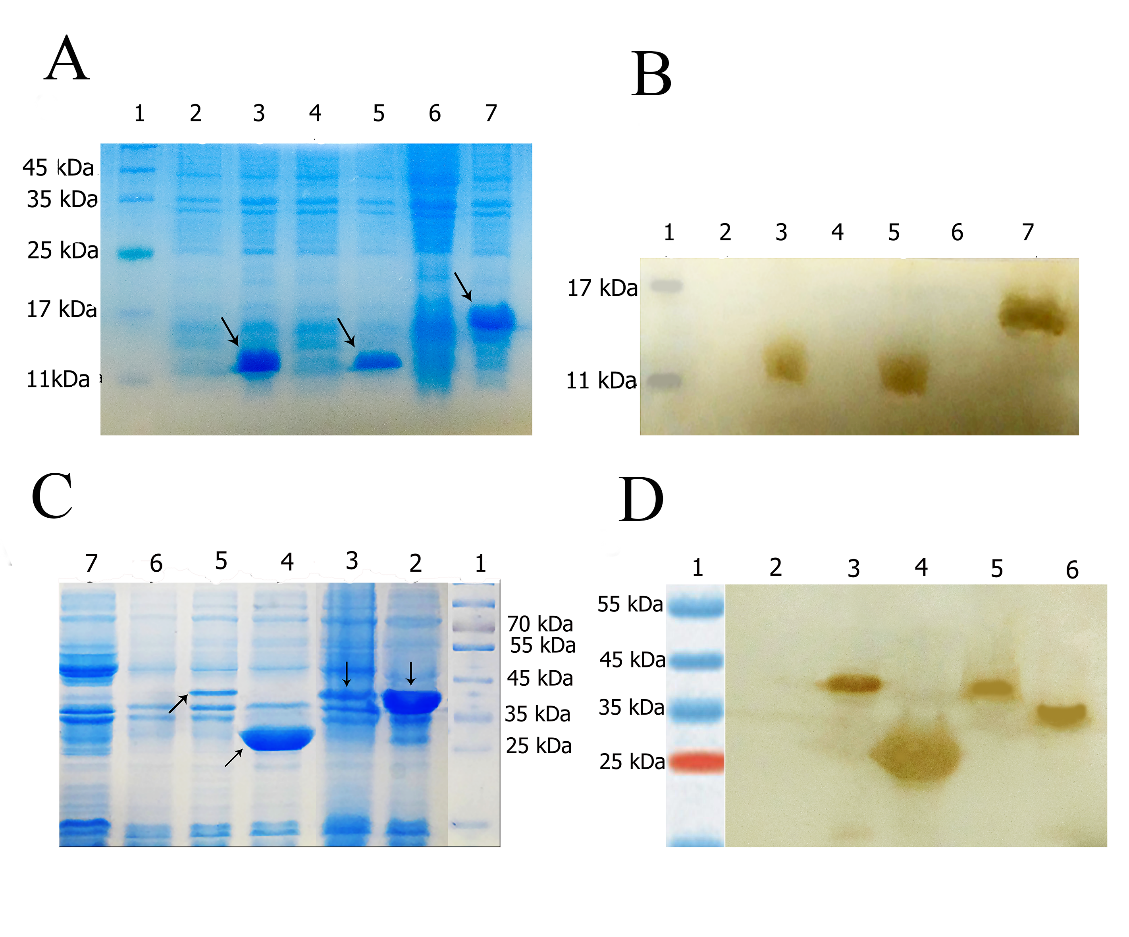


**Fig 2.** SDS-PAGE and western blotting of the expressed L2 dual-typed fusion peptides and L2 proteins amino acids 11-200. (A) and (B) SDS-PAGE and western blotting of the expressed dual-typed tandem repeat fusion peptides, respectively. Lanes in (A) and (B): 1; molecular weight marker, 2, 4, 6; un-induced cell lysates of the *E. coli* BL-21 (DE3) cells harboring pET-17 (encoding L2 17-36 ×3), pET-69 (encoding L2 69-81 ×3) and pET-88 (encoding L2 11-88 ×1), respectively. Lanes 3, 5, 7; Induced cell lysates of *E. coli* cells harboring the same constructs, respectively. (C) and (D) Expression of L2 11-200 residues from HPV-16, HPV-18, HPV-31 and HPV-45 in *E.coli* BL-21 (DE3) were confirmed by SDS-PAGE and western blot analysis, respectively. Lanes in (C): 1; molecular weight marker, 2, 3, 4, 5; the induced cell lysates of *E. coli* harboring pET-HPV45, pET-HPV31, pET-HPV18 and pET-HPV16, respectively. Lanes in (D): 1; molecular weight marker, 3, 4, 5, 6; the induced cell lysates of E. coli harboring pET-HPV45, pET-HPV31, pET-HPV18 and pET-HPV16, respectively. Lanes 6 and 7 in (C) and 2 in (D) represent un-induced cell induced cell lysates of E. coli host as negative control. Expressed bands are indicated by arrows in SDS-PAGEs. Please refer to the text for further explanations about protein sizes.
